# First-line therapy of osimertinib with chemotherapy in Chinese patients with EGFR mutation–positive non–small cell lung cancer: protocol for a multicenter, prospective, observational study

**DOI:** 10.3389/fonc.2025.1625714

**Published:** 2025-09-05

**Authors:** Bo Zhang, Fangling Ning, Xiaomei Liu, Na Li, Pingfei Wang, Wenxiu Yao, Baohui Han, Qiming Wang, Hua Zhong

**Affiliations:** ^1^ Department of Respiratory and Critical Care Medicine, Shanghai Chest Hospital, Shanghai, China; ^2^ Department of Oncology, Binzhou Medical University Hospital, Binzhou, Shandong, China; ^3^ Department of Oncology, The First Affiliated Hospital of Jinzhou Medical University, Jinzhou, Liaoning, China; ^4^ Department of Oncology Center, Suining Central Hospital, Sichuan, China; ^5^ Department of General Respiratory, Dazhou Central Hospital, Sichuan, China; ^6^ Sichuan Clinical Research Center for Cancer, Sichuan Cancer Hospital & Institute, Sichuan Cancer Center, Affiliated Cancer Hospital of University of Electronic Science and Technology of China, Chengdu, China; ^7^ Department of Oncology, The Affiliated Cancer Hospital of Zhengzhou University & Henan Cancer Hospital, Zhengzhou, Henan, China

**Keywords:** non-small cell lung cancer, epidermal growth factor receptor, tyrosine kinase inhibitor, osimertinib, real-world study

## Abstract

Epidermal growth factor receptor (EGFR) gene mutations are the most common oncogenic-genomic driver in non-small cell lung cancer (NSCLC), with highest prevalence (30%-50%) in Asian patients. Osimertinib, a third-generation EGFR tyrosine kinase inhibitor (EGFR-TKI) is the preferred first-line (1L) treatment for patients with NSCLC harboring EGFR-TKI- sensitizing mutations (exon 19del and/or exon 21 L858R). The FLAURA2 study suggested that combining chemotherapy with osimertinib treatment significantly improved progression-free survival (PFS) by approximately 9 months compared with osimertinib monotherapy among patients with EGFR-mutated advanced NSCLC. However, there is a lack of real-world efficacy and safety data for osimertinib in combination with chemotherapy, including the selection of different chemotherapy treatment patterns in real-world settings. This multicenter, prospective, observational study will recruit approximately 700 adult Chinese patients with histologically or cytologically documented non-squamous, stage IIIB/IIIC/IV NSCLC, EGFR sensitive mutation (exon 19del or 21 L858R, either alone or in combination with other EGFR mutations) and Eastern Cooperative Oncology Group (ECOG) performance status of 0 to 2 will be eligible to enroll in the study. Eligible patients will receive osimertinib plus chemotherapy as the 1L therapy based on the physician’s medical assessment. The primary endpoint is the median real-world PFS (rwPFS) as assessed by the investigator. Secondary endpoints include treatment pattern and dose intensity of 1L therapy, response rate, duration of response (DoR), overall survival (OS), and safety. Exploratory endpoints include second progression on a subsequent treatment (PFS2), time to treatment discontinuation (TTD), and time to first subsequent treatment (TFST). The central nervous system (CNS) response rate, CNS PFS, subsequent treatment pattern, progression patterns, and the genomic profile in the patients who had disease progression on receiving 1L osimertinib plus chemotherapy. The results of the proposed study aim to contribute evidence on the effectiveness and safety of osimertinib plus chemotherapy with diverse treatment patterns in Chinese patients with advanced NSCLC in real-world settings and support the clinical application of osimertinib-chemotherapy.

**Trial Registration:**

ClinicalTrials.gov, identifier NCT06376084 (Date of registration: 17-04-2024).

## 1 Introduction

In China, lung cancer is the most common cancer as well as the first leading cause of cancer deaths in 2016, with an estimated 828,100 new cancer cases (20.4%) and 657,000 cancer deaths (27.2%) (1). Patients with non-small cell lung cancer (NSCLC) have a poor 5-year survival: approximately 10% to 18% for all stages and less than 5% for those diagnosed at a late stage (2). Approximately 30% to 40% of patients in Asia have epidermal growth factor receptor (EGFR) mutated-positive NSCLC, and it can be up to 64.5% in the case of adenocarcinoma (3–5).

EGFR tyrosine kinase inhibitors (EGFR-TKIs) are the standard first-line (1L) treatment in advanced *EGFR*-mutant NSCLC possessing Ex19del or 21 L858R mutants (6). Osimertinib is the first FDA- and EMA-approved irreversible third-generation EGFR-TKI that inhibits EGFR-TKI sensitizing and T790M resistance mutations (7). In the phase III FLAURA trial, osimertinib demonstrated a significantly longer progression free survival (PFS) and an overall survival (OS) efficacy, along with superior safety outcomes, compared with the first-generation EGFR-TKIs such as erlotinib or gefitinib (7).

Osimertinib replaced the first-generation EGFR-TKI and became the new standard treatment option as the 1L therapy. However, despite demonstrating the promising results by osimertinib as a 1L therapy, the vast majority of patients are expected to develop resistance. Thus, there remains a significant unmet medical need for new treatment options for patients in this disease setting. In phase II and III trials, gefitinib demonstrated better efficacy when combined with carboplatin-based therapy compared with gefitinib alone. In the phase II OPAL study, osimertinib combined with platinum-pemetrexed chemotherapy showed promising results, by achieving a median objective response rate (ORR) of 90.9% and a median PFS of 31 months. This combination therapy was well tolerated and had a manageable safety profile with no new safety signals detected.

Based on these findings, it was hypothesized that the combination therapy of osimertinib with platinum-based therapy might have better clinical outcomes in patients with advanced NSCLC as compared with osimertinib monotherapy (7–9). The global phase III FLAURA2 trial further explored osimertinib with chemotherapy in patients with EGFR Ex19del and/or exon 21 L858R mutations who had not received prior systemic therapy for advanced NSCLC. The trial confirmed a significantly longer PFS with combination therapy compared with osimertinib monotherapy. In addition, interim OS results suggested a positive trend for osimertinib plus chemotherapy (hazard ratio [HR], 0.75; 95% confidence interval, 0.57-0.97), with consistent results across the prespecified subgroups (7). Based on the results, osimertinib with platinum-based chemotherapy was considered to be a preferred 1L treatment in patients with EGFR-mutated advanced or metastatic NSCLC.

However, there are limited data on the various treatment patterns—such as chemotherapy regimens, the number of induction chemotherapy cycles, duration of chemotherapy maintenance, and chemotherapy dose intensity, as well as their impact on clinical outcomes when osimertinib is combined with chemotherapy as a 1L treatment in real-world settings.

The proposed study intends to conduct a prospective analysis of data from approximately 700 patients who will receive this combination therapy as a 1L treatment for locally advanced or metastatic EGFR-mutated NSCLC in a real-world context.

## 2 Methods

### 2.1 Study design

**Figure 1:**
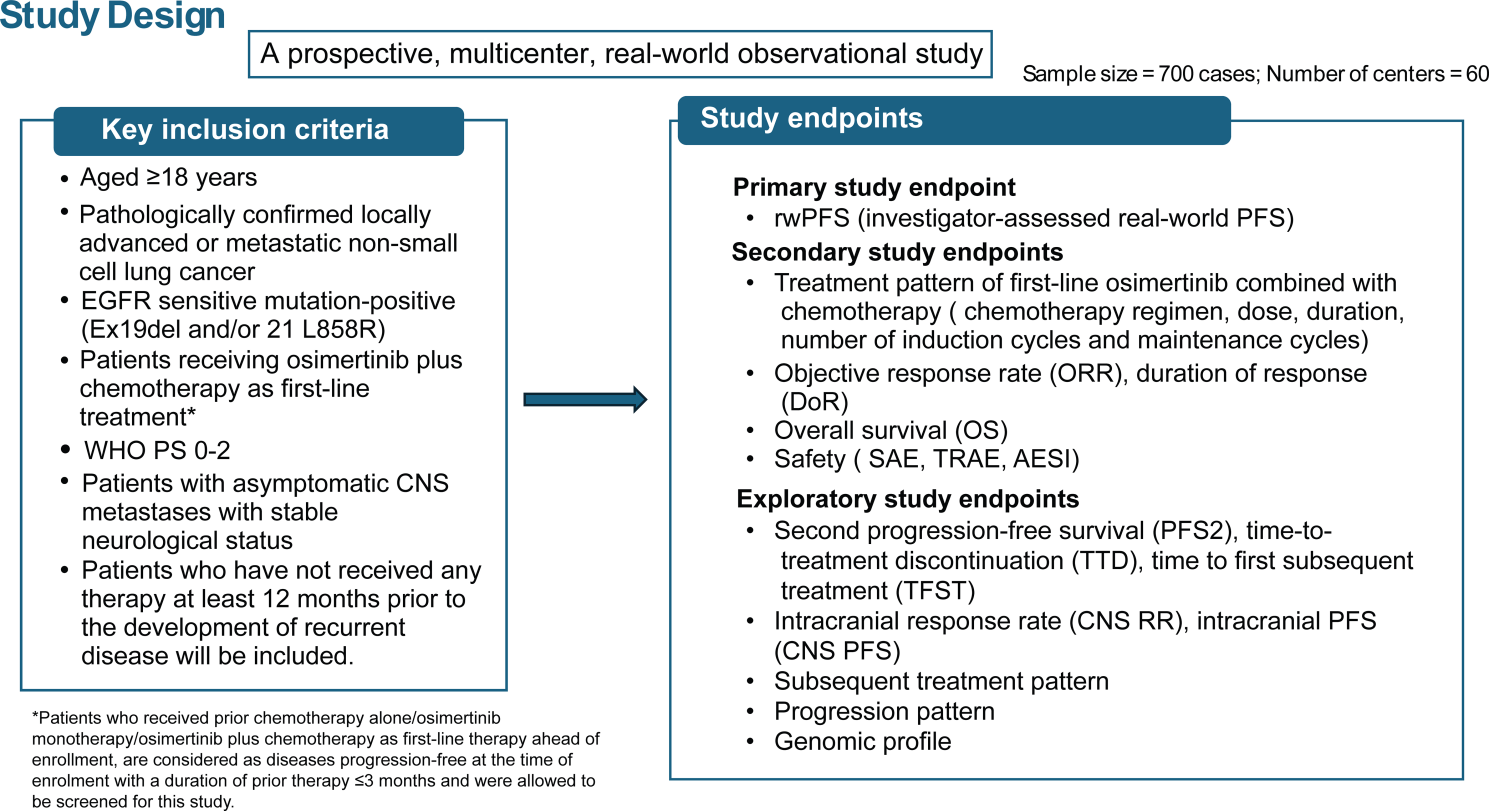
Study design.

This is a prospective, multicenter, observational, real-world study that will be conducted across 60 centers in China to determine the effectiveness and safety of 1L osimertinib combined with chemotherapy for the treatment of locally advanced or metastatic, EGFR mutation-positive NSCLC. The enrollment of all patients, treatment schedule, follow-up, and data collection will be conducted according to the physician’s medical assessment (**Figure 1**). The sample size of the study will be approximately 700 patients. All analysis will be based on full analysis set (FAS), which will include all patients who have taken at least one dose of osimertinib plus chemotherapy as 1L treatment. The central nervous system (CNS) FAS (cFAS) will be a subset of the FAS population and will include all patients who have undertaken a brain scan and were identified as having CNS metastases at baseline.

### 2.2 Data source

There will be a screening period of up to 28 days before the enrollment of eligible patients. Clinical outcomes will be assessed at the recommended treatment visit every 12 weeks until the discontinuation of the treatment or withdrawal of the patient from the study. After the recruitment of the last patient, there will be a follow-up period of approximately 36 months. Data from eligible patients will be collected during this period until a sufficiently mature dataset of approximately 60% on PFS is obtained. In addition, the safety data will be collected from the beginning of the 1L treatment and will be continued throughout the treatment period and during the safety follow-up.

### 2.3 Study patients

In this study, patients aged ≥18 years, with locally advanced or metastatic NSCLC with EGFR sensitive mutation-positive (Ex19del and/or 21 L858R), receiving osimertinib plus chemotherapy 1L treatment (in patients who received prior chemotherapy alone/osimertinib monotherapy/osimertinib plus chemotherapy as 1L therapy ahead of enrollment, are considered as disease progression-free at the time of enrollment with a duration of prior therapy ≤3 months), patients with asymptomatic CNS metastases with stable neurological status and who have not received any therapy at least 12 months prior to the development of recurrent disease will be included. Inclusion and exclusion criteria for patient enrollment is presented in **Table 1**.

**Table 1:** Inclusion and exclusion criteria.

Inclusion criteria	Exclusion criteria
18 years of age or older	Patients with spinal cord compression and symptomatic brain metastases.
Patients with confirmed NSCLC histologically or cytologically or demonstrated with NSCLC of mixed histology.	Patients with past medical history of ILD, drug-induced ILD, radiation pneumonitis that required steroid treatment, or any evidence of clinically active ILD.
Patients with locally advanced (stage IIIB or IIIC) or metastatic (stage IV) NSCLC and had not previously undergone curative surgery or chemoradiation; confirmed EGFR (Ex19del and/or 21 L858R) mutation.	Patients with severe or uncontrolled systemic diseases, including uncontrolled hypertension and active bleeding diatheses, etc.
Patients with a World health organization performance status of 0 to 2 at screening with no clinically significant deterioration in the previous 2 weeks.	Patients on any banned substance. Recruitment date will be from July 2024 to February 2028.
Patients who have been previously treated with osimertinib plus chemotherapy as first-line treatment or chemotherapy alone or osimertinib monotherapy based on physician’s medical assessment and the patients should be in disease-free progression stage with duration of prior therapy ≤3 months.	
Patients with asymptomatic CNS metastases should have completed the definitive therapy, neurological stable, and will not be receiving steroids for at least 2 weeks before commencing the trial treatment.	
Prior adjuvant and neo-adjuvant therapies (chemotherapy, radiotherapy, immunotherapy, biologic therapy, and investigational agents), or definitive radiation/chemoradiation with or without regimens including immunotherapy, biologic therapies, investigational agents will be allowed provided that they should have completed minimum 12 months before the disease recur.	

CNS, central nervous system; ILD, interstitial lung disease; NSCLC, non-small cell lung cancer.

### 2.4 Outcomes

#### 2.4.1 Primary outcome

The primary objective of this study will be to evaluate the PFS in a real-world setting (rwPFS), defined as the time from initiation of the 1L treatment until the disease progression or death assessed by the investigator. A subgroup analysis for rwPFS will be conducted when applicable with at least 10 events: (1) gender; (2) age at screening (<65 or ≥65 years); (3) smoking history; (4) WHO performance status (PS) (0, 1 or 2); (5) EGFR mutation type (exon 19 del and/or 21 L858R); (6) CNS status at baseline (Yes, No or Unknown); (7) chemotherapy regimens (carboplatin/cisplatin with pemetrexed or other regimens, or without carboplatin/cisplatin); (8) Number of induction chemotherapy cycles (≤4, >4, ≤6, and >6 cycles); (9) duration of chemotherapy maintenance (≤6, 6–12, and >12 months), and (10) dose intensity of chemotherapy.

During the treatment follow-up period, efficacy data will be recommended to collect from the start of the treatment for every 12 weeks until objective disease progression as defined by the investigator, withdrawn or another discontinuation criterion is met.

#### 2.4.2 Secondary endpoint

The secondary endpoint included the assessment of treatment patterns in real-world settings such as chemotherapy regimen, dose, duration, number of induction cycles and maintenance cycles. The different chemotherapy regimens will be categorized as carboplatin/cisplatin combined with pemetrexed or other regimen, or chemotherapy without carboplatin/cisplatin. Other secondary efficacy endpoints include ORR, which is defined as the proportion of patients with complete response or partial response. Furthermore, the duration of response (DoR) and OS will also be assessed.

#### 2.4.3 Safety endpoint

Safety endpoints include the incidence of serious adverse events (SAEs), treatment-related adverse events (TRAEs), and adverse events of special interest (AESI) (including pneumonitis, cardiac failure, and hematologic toxicities) and abnormal laboratory findings.

#### 2.4.4 Exploratory endpoint

The exploratory endpoints include second progression on a subsequent treatment (PFS2), time to treatment discontinuation (TTD). TTD is defined as the duration of time from which a patient started receiving osimertinib and chemotherapy till discontinuation of therapy for any reason. Other exploratory endpoints include time to first subsequent treatment (TFST), CNS PFS, subsequent treatment pattern, and progression patterns, genomic profile including but not limited to mutations in amplifications and expression of EGFR, TP53, HER2, MET, and relevant pathway genes after failure of 1L osimertinib plus chemotherapy.

### 2.5 Statistical analyses

All time to event endpoints will be summarized using Kaplan-Meier estimates of the median event time and quartiles together with their 95% confidence intervals (CIs). The CNS response rate will be summarized descriptively, and the Clopper–Pearson 95% CIs will be provided. Treatment patterns, progression patterns, and incidence of new CNS metastases will be summarized descriptively. Descriptive summary will be provided for TRAE, SAE, and AESI (interstitial lung disease [ILD], including pneumonitis, cardiac failure, and hematological toxicities) and exploratory endpoints. Safety endpoints will be graded by the investigators using Common Terminology Criteria for Adverse Events (CTCAE) version 5.0 and coded using the Medical Dictionary for Regulatory Activities (MedDRA).

### 2.6 Sample size calculation

This is a single-arm, observational study without a pre-defined study hypothesis test with no power calculation required and no formal sample size calculation to be done. However, a total of approximately 700 patients will be treated by osimertinib with chemotherapy. An illustration of the sample size justification is presented in **Table 2**.

**Table 2:** An illustration of the precision with varying sample size.

Sample size	Median rwPFS (months)	95% CI	Precision (Half of 95% CI) (months)
800	26	23.3–28.8	2.8
700	26	23.2–29.0	2.9
600	26	22.9–29.3	3.2

rwPFS, real-world progression free survival.

## 3 Discussion

To the best of our knowledge, this will be the first multicenter, prospective, observational study aimed to evaluate the safety and effectiveness of osimertinib with chemotherapy in Chinese patients with metastatic NSCLC harboring an EGFR exon 19 deletion and/or exon 21 L858R mutation in a real-world setting. The FLAURA2 study was conducted to investigate the effectiveness and safety of osimertinib with or without platinum plus pemetrexed chemotherapy considering the additive anti-tumor activity of chemotherapy (10). The results from this trial demonstrated prolonged PFS (25.5m vs 16.7m, HR 0.62) and OS (not reached vs. 36.7m, HR 0.75;41% maturity) benefit trend with the combination therapy as compared with osimertinib monotherapy in patients with EGFR-mutated advanced NSCLC. Although combination therapy has potential, the efficacy and safety profile of this approach in real-world clinical settings are yet to be fully established, warranting additional research. As opposed to the strict inclusion and exclusion criteria and predefined outcomes in clinical trials, real-world data reflects a broader population with various comorbidities and outcomes and can complement trials with larger volumes of data over longer time periods (11). In the FLAURA2 study, patients with PS scores of 0–1 were enrolled, but this study also included patients with PS scores of 0–2 to assess the efficacy and safety of osimertinib in combination with chemotherapy, in order to meet the clinical treatment needs of such patients in the real world. The choice of chemotherapy regimen in FLAURA 2 study required the use of 4 cycles of pemetrexed and platinum-based treatment during the induction phase, followed by maintenance therapy with pemetrexed in combination with osimertinib until disease progression. The results showed that patients in the combined treatment group received a median of 12 cycles (range 1-48) of pemetrexed treatment, and 76% of patients completed 4 cycles of platinum-based chemotherapy. Moreover, the FLAURA2 study, conducted globally, in the Chinese population the drug exposure time for osimertinib and chemotherapy was longer compared to the global population, and the discontinuation rate due to adverse events in the combined treatment group in the Chinese cohort was relatively low (29% vs. 48%). Considering factors such as toxicity, cost, patient burden and compliance, it is challenging for patients in the real world to complete the chemotherapy drugs, dosages, and cycles as required by the registration study. Therefore, the study explores different chemotherapy regimens in a real-world setting, such as not restricting the types of clinical drugs chosen by physicians and adjusting chemotherapy drugs and cycles according to the physician’s judgment, encompassing both induction and maintenance phases, with the goal of optimizing treatment outcomes for patients. Selecting the appropriate treatment plan after resistance to 1L EGFR-mutated advanced NSCLC therapy to maximize the extension of patient survival is a critical clinical need. This involves exploring subsequent treatment strategies such as oligoprogression, extensive progression, and the incidence of new CNS metastases. Additionally, it includes assessing the situation of genetic testing conducted in China in post 1L osimertinib plus chemotherapy resistance setting, the real-world genomic profile after 1L osimertinib plus chemotherapy failure, and the impact of various post-resistant treatment options on PFS2 and OS. Our proposed study is the first real-world study to enrich evidence supporting the clinical benefit of using osimertinib with chemotherapy as 1L for the treatment of EGFR mutation-positive in Chinese patients with locally advanced or metastatic NSCLC. However, our proposed study has certain limitations. The inclusion criterion - restricting enrollment to patients who received prior osimertinib combined with chemotherapy for a duration of ≤3 months and remain disease progression free at screening, could result in the systematic exclusion of patients who experienced rapid disease progression or death during the initial treatment window. A series of sensitivity analyses will be pre-specified to assess the robustness of our findings in the statistical analysis plan. These include a landmark analysis in which only patients who remain progression-free at a predefined time point (e.g. 3 months from treatment initiation) will be included in survival analysis from that point forward. This approach allows us to evaluate the consistency of treatment outcomes while accounting for the effect of the eligibility window.

## Glossary

AESI: Adverse events of special interest
CIs: Confidence intervals
CNS: Central nervous system
cFAS: CNS full analysis set
CTCAE: Common Terminology Criteria for Adverse Events
DoR: Duration of response
ECOG: Eastern Cooperative Oncology Group
EGFR: Epidermal growth factor receptor
EGFR-TKI: EGFR tyrosine kinase inhibitor
FAS: Full analysis set
1L: First-Line
HR: Hazard ratio
ILD: Interstitial lung disease
MedDRA: Medical Dictionary for Regulatory Activities
NSCLC: Non-small cell lung cancer
ORR: Objective response rate
OS: Overall survival
PFS: Progression-free survival
PFS2: Second progression on a subsequent treatment
rwPFS: Real-world PFS
SAEs: Serious adverse events
TFST: Time to first subsequent treatment
TRAEs: Treatment-related adverse events
TTD: Time to treatment discontinuation
